# An epidemiological and molecular study regarding the spread of vancomycin-resistant *Enterococcus faecium* in a teaching hospital in Bogotá, Colombia 2016

**DOI:** 10.1186/s12879-019-3877-7

**Published:** 2019-03-15

**Authors:** Nancy Carolina Corredor, Carolina López, Paula Andrea Aguilera, Lina María Prieto, Giovanni Rodríguez-Leguizamón, Aura Lucía Leal, María Victoria Ovalle-Guerro, Juan Mauricio Pardo-Oviedo, Claudia Elena Chica, Manuel Alfonso Patarroyo

**Affiliations:** 1 Universidad del Rosario – Hospital Universitario Mayor Méderi, Bogotá, Colombia; 2 Masters’ Programme in Epidemiology, Universidad del Rosario, Bogotá, Colombia; 3 Molecular Biology and Immunology Department, Fundación Instituto de Inmunología de Colombia (FIDIC), Carrera 50#26-20, Bogotá, Colombia; 4 PhD Programme in Biomedical and Biological Sciences, Universidad del Rosario, Bogotá, Colombia; 5 School of Medicine and Health Sciences, Universidad El Bosque, Bogotá, Colombia; 6 School of Medicine and Health Sciences, Universidad del Rosario, Bogotá, Colombia; 7 Microbiology Department, Medical School, Universidad Nacional de Colombia, Bogotá, Colombia; 8 Clinical lab, Compensar, Bogotá, Colombia

**Keywords:** Vancomycin-resistant *Enterococcus faecium*, Outbreak surveillance, Antimicrobial resistance, Time–place–sequence algorithm

## Abstract

**Background:**

*Enterococcus faecium* is ranked worldwide as one of the top ten pathogens identified in healthcare-associated infections (HAI) and is classified as one of the high priority pathogens for research and development of new antibiotics worldwide. Due to molecular biology techniques’ higher costs, the approach for identifying and controlling infectious diseases in developing countries has been based on clinical and epidemiological perspectives. Nevertheless, after an abrupt vancomycin-resistant *Enterococcus faecium* dissemination in the Méderi teaching hospital, ending up in an outbreak, further measures needed to be taken into consideration. The present study describes the vancomycin-resistant *Enterococcus faecium* pattern within Colombian’s largest installed-bed capacity hospital in 2016.

**Methods:**

Thirty-three vancomycin-resistant *Enterococcus faecium* isolates were recovered during a 5-month period in 2016. Multilocus variable-number tandem-repeat analysis was used for molecular typing to determine clonality amongst strains. A modified time-place-sequence algorithm was used to trace VR*Efm* spread patterns during the outbreak period and estimate transmission routes.

**Results:**

Four clonal profiles were identified. Chronological clonal profile follow-up suggested a transitional spread from profile “A” to profile “B”, returning to a higher prevalence of “A” by the end of the study. Antibiotic susceptibility indicated high-level vancomycin-resistance in most isolates frequently matching v*anA* gene identification.

**Discussion:**

Transmission analysis suggested cross-contamination via healthcare workers. Despite epidemiological control of the outbreak, post-outbreak isolates were still being identified as having outbreak-related clonal profile (A), indicating reduction but not eradication of this clonality. This study supports the use of combined molecular and epidemiological strategies in an approach to controlling infectious diseases. It contributes towards a more accurate evaluation of the effectiveness of the epidemiological measures taken regarding outbreak control and estimates the main cause related to the spread of this microorganism.

**Electronic supplementary material:**

The online version of this article (10.1186/s12879-019-3877-7) contains supplementary material, which is available to authorized users.

## Background

Microorganisms having antimicrobial resistance (AMR) patterns are a threat regarding current epidemiological transition of diseases worldwide (from communicable to non-communicable ones). It has been estimated that infectious diseases could re-emerge as the main cause of mortality worldwide by 2050 [1–3].

*Enterococcus faecium* (*Efm*) has gained particular importance amongst healthcare-associated infections (HAIs) due to its presence worldwide and ability to persist for long periods in healthcare-related settings [4–6]. Moreover, its high recombination rate and horizontal gene transfer ability have allowed it to easily acquire resistance phenotypes [7, 8].

Vancomycin-resistant *E. faecium* (VR*Efm*) was first isolated in Europe and the USA by the end of the 1980s and seems to have appeared as a consequence of avoparcin (growth promoter) misuse in livestock and antibiotic overuse in hospital settings. *Enterococcus* sp. is ranked as the fifth most frequently-identified HAI-related pathogen [3, 9]. It is an *ESKAPE* bacteria, i.e. those which “escape” from most currently available antibiotics. *Efm* accounted for 3.7% of HAIs according to the summary of data reported to CDC’s national automated biosurveillance system regarding HAI-associated antimicrobial-resistant pathogens from 2011 to 2014 in the USA, a tendency which has also been described in Colombia [10].

The WHO recently included vancomycin-resistant *Enterococci* (VRE) as a priority pathogen for R&D regarding new public healthcare therapeutic options [11–13]. VR*Efm* infection has a significant impact on health and increases healthcare-related costs as additional therapeutic efforts are required. These efforts, mainly related to the pathogen’s resistance to almost all therapeutic options currently available, consist in strengthening isolation precautions to minimise spread of infection and implementing combined last-resource broad-spectrum therapies [14–16]. Most contact precaution signs amongst multi-drug resistance organisms in New York hospitals in 2010 were indicated for VR*Efm* and methicillin-resistant *Staphylococcus aureus* isolation [17]. VRE acquisition rate ranged from 1.9 to 37% amongst hospitalised patients (depending on length of exposure and proximity) and from 0.4 to 11.8% in the community [18–21].

The first VR*Efm* isolate in Colombia was reported in 1998 in Medellín; since then, it has spread throughout the country [22]. The number of VR*Efm* isolates reported by the Colombian National Institute of Health from 2014 to 2016 has been variable, the highest incidence occurring in 2015 [10, 23–26]. VR*Efm* represented 22.9% (*n* = 774), 27.6% (*n* = 1278) and 24.3% (*n* = 905) of the *Efm* isolates respectively from 2014 to 2016. The first *Efm* to be documented in the Méderi teaching hospital was identified in January 2001, followed by the first VR*Efm* in August 2001 and an average of up to 1 related HAI per month until 2016. The first VR*Efm* outbreak was identified in May 2016 resulting from the abrupt spread of this microorganism as one of the major HAI-related bacteria in the hospital.

The present study was aimed at describing the VR*Efm* pattern in 2016, including a molecular study of strains recovered from May to September 2016 in the Méderi teaching hospital. A modified time-place-sequence (TPS) algorithm [27] was used to estimate transmission routes during the VR*Efm* outbreak (May 2016).

## Methods

### Study design

This was an observational, cross-sectional descriptive study. It was aimed at describing nosocomial VR*Efm* pattern within the hospital, integrating epidemiological and molecular typing methods through a TPS algorithm. The study lasted 5 months, from May to September 2016 (i.e. enabling the study of outbreak and post-outbreak strains). Case definition followed CDC HAI criteria [28].

### Clinical setting and data collection

**Fig. 1:**
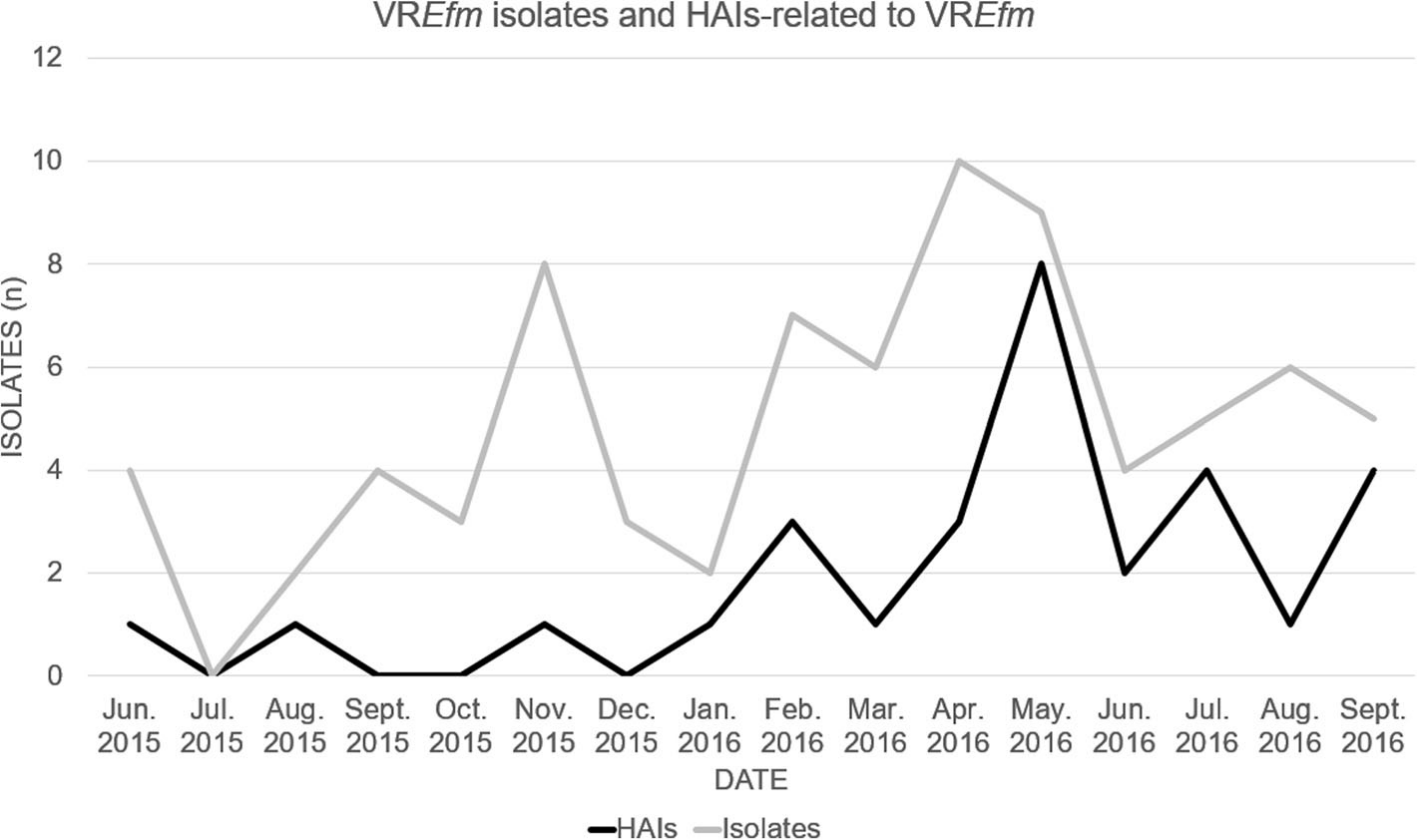
VR*Efm* isolates and VR*Efm*-related HAI. The Figure shows the number of VR*Efm* isolates and VR*Efm*-related HAI in the Méderi hospital from June 2015 to September 2016

The study took place in an 802-bed tertiary-care teaching hospital, in Bogotá, Colombia. The hospital is divided into 2 healthcare facilities and the VR*Efm* outbreak had place in largest one. It has a 506-bed capacity in adult general wards; there are 118 beds in its adult intensive care unit (ICU), 10 beds in its coronary care unit (CCU), 22 beds in its adult intermediate care unit (IMCU) and 25 beds in its neonatal intensive care unit (NICU). *Efm* has been considered an endemic microorganism in the Méderi teaching hospital since its first isolation in 2001; however, VR*Efm* caused few infections until 2016. After a sudden increase in VR*Efm* isolates in February 2016 (Fig. 1), the hospital’s Epidemiology Department started a healthcare–associated infection outbreak investigation and a VR*Efm*-related outbreak was confirmed for May 2016. Nevertheless, some cases from the end of April and first days of June were also included within the outbreak investigation according to the analysis made by the hospital’s epidemiology department. Outbreak confirmation was based on the index of VR*Efm-*related infections compared to the number of patients discharged per month, in at least 24 months. If this index was above the third standard deviation (SD) an outbreak was confirmed following criteria established by the Bogotá District Health Secretariat [29]. These criteria include:

1. An increased incidence of an infection, higher than expected, and/or
2. The occurrence of an increase in the number of cases, in relation to an index case, and/or
3. The onset of an emergent (first) case by a microorganism or modifications in the antimicrobial resistance pattern in a specific place, time and population.

**Fig. 2:**
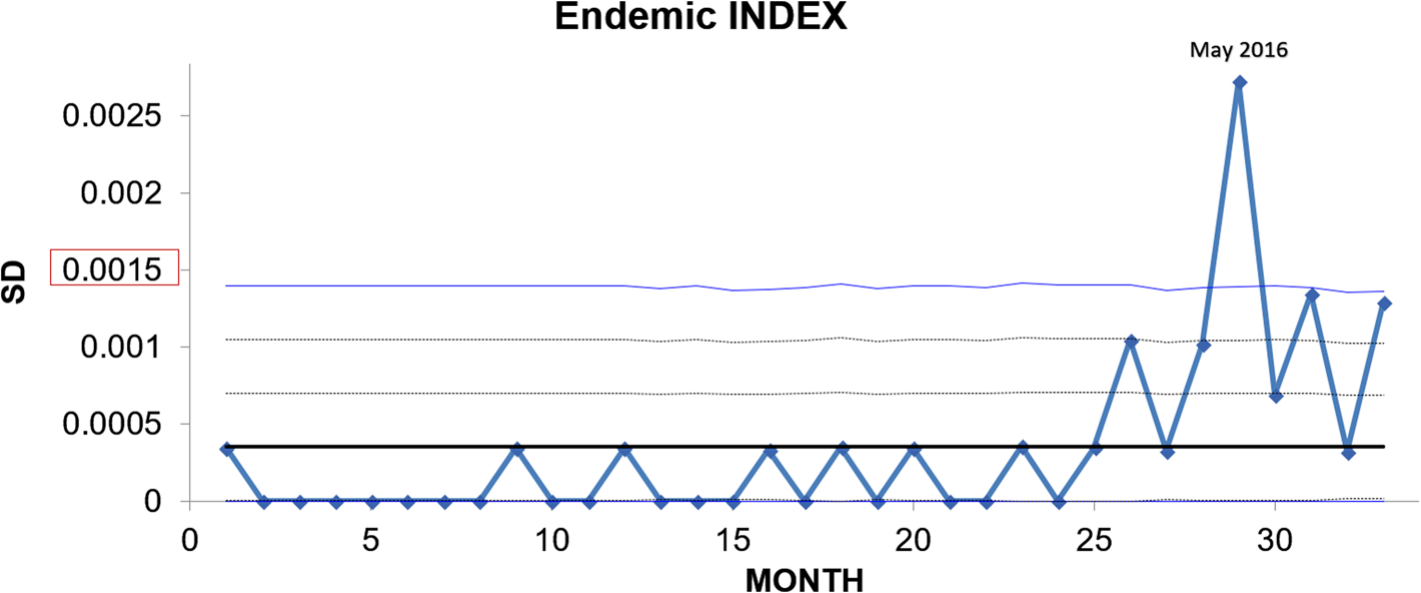
Endemic Index. An index amongst the VR*Efm-*related infections and number of discharged patients per month, in at least 24 months. Zero (0) in months corresponds to January 2014, and 30 to June 2016. If this index is above the third standard deviation (SD), an outbreak is confirmed, according to the Bogotá District Health Secretariat [29]

This index also confirmed outbreak control in June 2016, following primary multidisciplinary interventions (Fig. 2). A post-outbreak study was made from July to September 2016.

### Microbiological identification

*Enterococcus faecium* species were identified by Vitek 2 system mass spectrometer (software version 1.02, bioMérieux). Isolates’ in vitro susceptibility to antimicrobial drugs was determined by automated Vitek 2XLS card, based on Clinical Laboratory Standards Institute (CLSI) 2016 [30] criteria for *Enterococcus*. The first confirmatory manual method for vancomycin-resistance identification involved the E-test gradient diffusion method. Additional manual microdilution to quantify minimum inhibitory concentrations (MICs) for vancomycin and teicoplanin resistance was made by the Universidad Nacional de Colombia’s Microbiology Department, using previously identified vancomycin-resistant *Enterococcus faecium* strains. *Enterococcus faecalis* ATCC 29212 (NCTC 12697) was used as control strain and CLSI M100-S24 was used for interpreting the results.

### *vanA* gene detection and molecular typing

*vanA* gene detection and molecular typing was performed for all 33 recovered strains; PCR was used for evaluating v*anA* detection. Primer sequences were based on the genes published for *Efm* [31, 32]. The reactions were performed with AmpliTaq Gold DNA polymerase hot-start enzyme with Buffer I (Applied Biosystems, Foster City, CA, USA); PCR fragments were visualised on 2% agarose gel.

Molecular typing involved VR*Efm* isolates being grown in LB broth overnight with dextrose supplement (5 g/L) at 37 °C with shaking. A Wizard Genomic DNA Purification Kit (Madison, Wisconsin, USA) was used for extracting DNA, following the gram-positive bacteria protocol; 120 μL lysozyme (10 mg/mL) was used for cell lysis. Variable-number tandem-repeat (VNTR) analysis was chosen for determining clonality; VNTR-1, VNTR-7, VNTR-8, VNTR-9 and VNTR-10 were amplified since there were no consistent and/or reproducible results for VNTR-2 (required for multilocus variable number tandem-repeat analysis (MLVA)) [33]. The 5 VNTR loci were processed as described by Top et al., for MLVA, with some minor modifications [34].

Briefly, VNTR-1 PCR conditions were modified, involving 30 cycles. A touchdown PCR was used for VNTR-7, VNTR-8, VNTR-9 and VNTR-10, using the same conditions described by Top et al., except for initial touchdown (TD) temperature for VNTR-9 which was 65 °C, decreasing to 55 °C. The reactions were performed in 10 μL volume with an AmpliTaq Gold DNA Polymerase hot-start enzyme with Buffer I (Applied Biosystems, Foster City, CA USA). PCR fragments were visualised on 2% (*w*/*v*) agarose gel using GelRed nucleic acid gel stain (Biotium). These 5 VNTRs’ grouping results defined our clonal profiles.

### Transmission analysis

A retrospective outbreak analysis was made, mainly for identifying transmission routes rather than the source(s) of infection. This perspective was considered, taking into account that we could not clearly identify the index case, due to *Enterococcus faecium* being classified as an endemic pathogen since its first isolation in 2001 and an increased number of isolates having been observed during the first months of 2016. Transmission analysis was then performed by adapting an algorithm previously described for a long-term outbreak of *Pseudomonas aeruginosa* in Germany from 2002 to 2015 [27], having successfully identified transmission routes. The modifications involved using VNTR analysis rather than whole genome sequencing (WGS) for assessing clonality. The outbreak involved 16 patients: 3 patients from April, 9 from May and 4 from June. All available outbreak strains (13 isolates) were included for this analysis since isolates from the first three patients could not be recovered.

Transmission was hypothetically considered if VR*Efm* had first been detected in patient “A” before VR*Efm* was first detected in patient “B”. Patients’ epidemiological tracking data and clonal profiles detected from outbreak isolates were combined to establish four criteria: criterion 1 was fulfilled if patients A and B were located in the same ward (24 h minimum overlap before VR*Efm* was first detected in patient B), criterion 2 was fulfilled if patient B was located in the same room which patient A had occupied a maximum 2 weeks before patient B, criterion 3 concerned both patient A and patient B having stayed in the same room (minimum 24 h overlap before VR*Efm* was detected in patient B) and criterion 4 concerned close genetic relatedness between VR*Efm* isolates from patients A and B.

Possible transmission was considered when criterion 1 was fulfilled**;** probable transmission was considered when either 2 or 3 were fulfilled, as well as fulfilment of 4 alone. Predicted transmission was determined when 4 was fulfilled for patients A and B in combination with any of the three epidemiological criteria (1, 2 or 3); if no criteria were fulfilled, the probability of transmission was considered unknown [27].

Estimator-predictor epidemiology significance pointed out some deficiencies regarding cleaning and disinfection. Possible transmission (criterion 1 – same ward) could have occurred via healthcare personnel (patients fulfilling this criterion never shared a room but the same ward, receiving medical attention from the same healthcare personnel). Probable transmission (criterion 2, 3 – same room or criterion 4 – genetic relatedness) could have occurred via direct contact or environmental contamination. Predictable transmission (combination of any epidemiological criterion and genetic relatedness) included the previous explanations.

## Results

Thirty-three non-duplicated VRE*fm* isolates were recovered over a 5-month period (May to September 2016) from 29 inpatients and another 4 from environmental surfaces. Thirteen of the 29 isolates from patients and the 4 environmental samples came from the outbreak; the remaining 16 isolates were obtained from patients during the post-outbreak period (July to September 2016) including infections and colonisations. Clinical samples were obtained from patients as follows: 18 from urine (62.1%), 7 from surgical wounds (21.4%), 3 from blood (10.34%) and one from orotracheal discharge (3.44%). Environmental surfaces’ samples were taken from 2 different patient bed-rails from general wards and an ICU infusion pump. Three of the 29 clinical isolates (10.3%) were considered to be community-acquired or having been acquired outside the healthcare setting.

### Outbreak setting and interventions

The Endemic Index confirmed that the outbreak occurred in May 2016, but patients from the end of April, May and the beginning of June were also included, according to the Epidemiology Department’s analysis. Different outbreak control strategies were introduced in line with Bogotá Territorial Health Department and Society for Healthcare Epidemiology of America (SHEA) recommendations for preventing healthcare-associated infections [29, 35] (Additional file 1: Figure S1).

The VR*Efm* outbreak involved 16 patients. Upon reanalysis made by the infectious diseases department by retrospectively verifying case definitions according to the CDC criteria [28], four patients ended up being classified as having been colonised. One clinical infection was identified as having been acquired before admission to Méderi (external sources). After 6 patients died and 4 deaths were estimated as being attributable to VR*Efm* infection, the VR*Efm* outbreak became controlled in June 2016 (just 2 VR*Efm*-related infections during this month) (Figs. 1 and 2). Nevertheless, post-outbreak assessment was pursued following national and international recommendations to assess outbreak intervention efficacy and verify outbreak control; 16 VR*Efm* isolates recovered from inpatients from June to September were preserved for these purposes.

Environmental samples were obtained as quality control for cleaning and disinfection during the outbreak; 33 healthcare surfaces were evaluated on June 2nd, 2016, including some healthcare providers’ hands, bed rails, infusion pumps, monitoring equipment, computer keyboards and bedside nurse call buttons. These cultures revealed VRE*fm* growth on three surfaces: 2 bed rails from different rooms in one general ward and one ICU infusion pump. Disinfection procedures were reinforced and followed-up on June 13th; two of the three surfaces proved negative and a VR*Efm* isolate was recovered from the remaining bed-rail on the fifth floor. This surface received a second follow-up on June 20th (no VR*Efm* isolation).

### Phenotypic antibiotic susceptibility

The 33 isolates were evaluated for antibiotic susceptibility but only the 29 clinical VR*Efm* isolates were included in such analysis. Automated MICs were determined for ampicillin, ciprofloxacin, high-load streptomycin, high-load gentamicin, linezolid, quinupristin-dalfopristin, tetracycline, vancomycin and teicoplanin. Manual microdilution was performed to determine vancomycin and teicoplanin MICs. All isolates (100%) were confirmed as vancomycin- and ciprofloxacin-resistant strains, susceptible to linezolid, high-load gentamicin and quinupristin-dalfopristin; all but one (i.e. 28 isolates) were identified as teicoplanin- and ampicillin-resistant. Twenty-four had resistance to high-load streptomycin and 21 were tetracycline-resistant. Four qualitative antibiotic susceptibility patterns were identified (Table 1); manual methods revealed 26 isolates (89.6%) as having high-level vancomycin resistance (MIC> 128 μg/dL) and 28 isolates (96%) as being teicoplanin-resistant. One isolate was susceptible to ampicillin and teicoplanin, an unusual pattern for this kind of bacteria [16, 36, 37].

**Table 1:** Qualitative susceptibility according to resistance (R) and susceptibility (S) patterns

Phenotypical profile	TEC	AMP	CIP	STH	GEH	LNZ	QDA	TCY	n	%
1	S	S	R	R	S	S	S	R	1	3.45
2	R	R	R	R	S	S	S	R	20	68.97
3	R	R	R	R	S	S	S	S	3	10.34
4	R	R	R	S	S	S	S	S	5	17.24

*TEC* teicoplanin, *AMP* ampicillin, *CIP* ciprofloxacin, *STH* streptomycin high load, *GEH* gentamicin high load, *LNZ* linezolid, *QDA* quinupristin-dalfopristin, *TCY* tetracycline. n: number of isolates expressing the target profile. %: percentage of isolates having the target phenotypical profile

### *vanA* gene detection

PCR identified the *vanA* gene in 26 of the 29 clinical isolates and in all 4 environment-recovered strains; *vanA* detection was related to medium and high-level vancomycin resistance (64 to > 512 MIC) and heterogeneous teicoplanin resistance (8 to < 256 MIC) [31, 36, 38, 39]. One of these 26 clinical isolates proving positive for the v*anA* gene was also identified as the only ampicillin- and teicoplanin-susceptible strain. Four isolates expressed a high-level vancomycin-resistance phenotype despite lack of *vanA* gene amplification (Table 2).

**Table 2:**
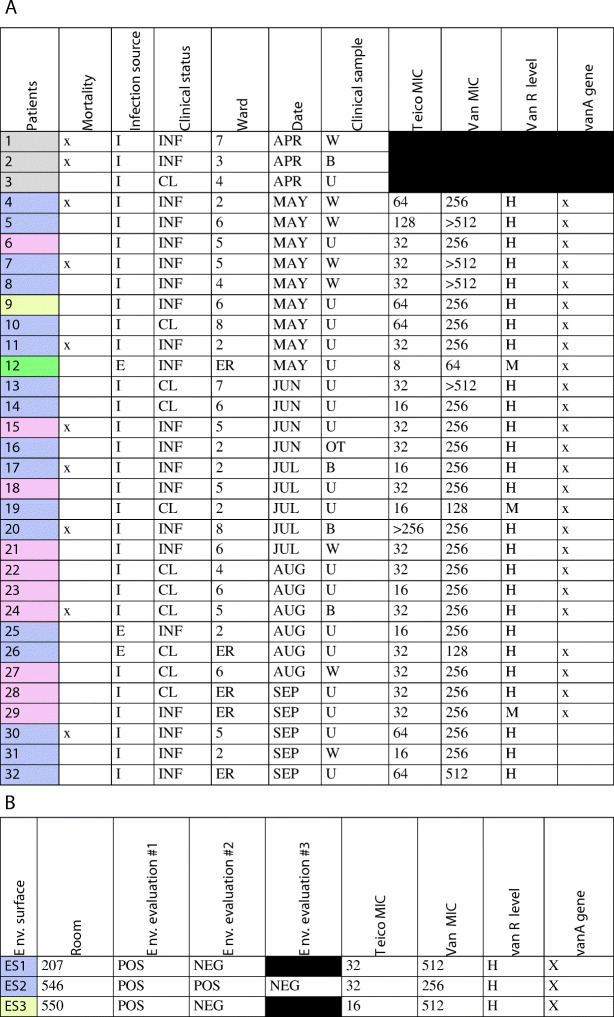
A and B Clonal profiles by VNTR-grouping related to epidemiological and microbiological data

**Colour pattern: purple** represents clonal profile “A”, **pink** profile “B”, **yellow** profile “C” and **green** profile “D”. **Patients:** “P” refers to a particular patient from whom the VR*Efm* strains were obtained according to chronological isolation. No molecular typing was performed for the first three outbreak isolates (grey). **Mortality:** death occurring during this period. **Infection source:** estimated location from where a patient acquired infection; I: institutional, E: external. **Clinical status**. INF: clinical infection, CL: bacterial colonisation. **Ward:** hospital wards where an isolate was obtained. ER: emergency room. Second floor (2) refers to ICU, 3 to IMCU, 4 to fourth floor, 5 to fifth floor, 6 to sixth floor, 7 to seventh floor and 8 eighth floor. **Date:** month when isolate was obtained. **Clinical sample** from which an isolate was recovered: surgical wound (W), urine (U), environmental surface (E), orotracheal sample (OT), blood (B). **Teico MIC:** teicoplanin MICs, according to manual microdilution. **Van MIC:** vancomycin MICs, according to manual microdilution**. Van R level -** vancomycin-resistance level, according to MIC, classified as high (H) and medium (M). ***vanA*** **gene:** identification of *vanA* gene, (x) *vanA* gene carrier. Table 2B. Environmental **surveillance. Environmental surface:** bed-rail (BR), infusion pump (IP). **Room:** rooms in which VR*Efm* was identified (207 in the ICU, 546–550 on the fifth floor). **Teico MIC:** teicoplanin MICs, according to manual microdilution. **Van MIC:** vancomycin MICs according to manual microdilution**. Van R level -** vancomycin-resistance level according to MICs, classified as high (H) and medium (M). ***vanA*** **gene:** identification of *vanA* gene, (x) *vanA* gene carrier

### Molecular typing

**Fig. 3:**
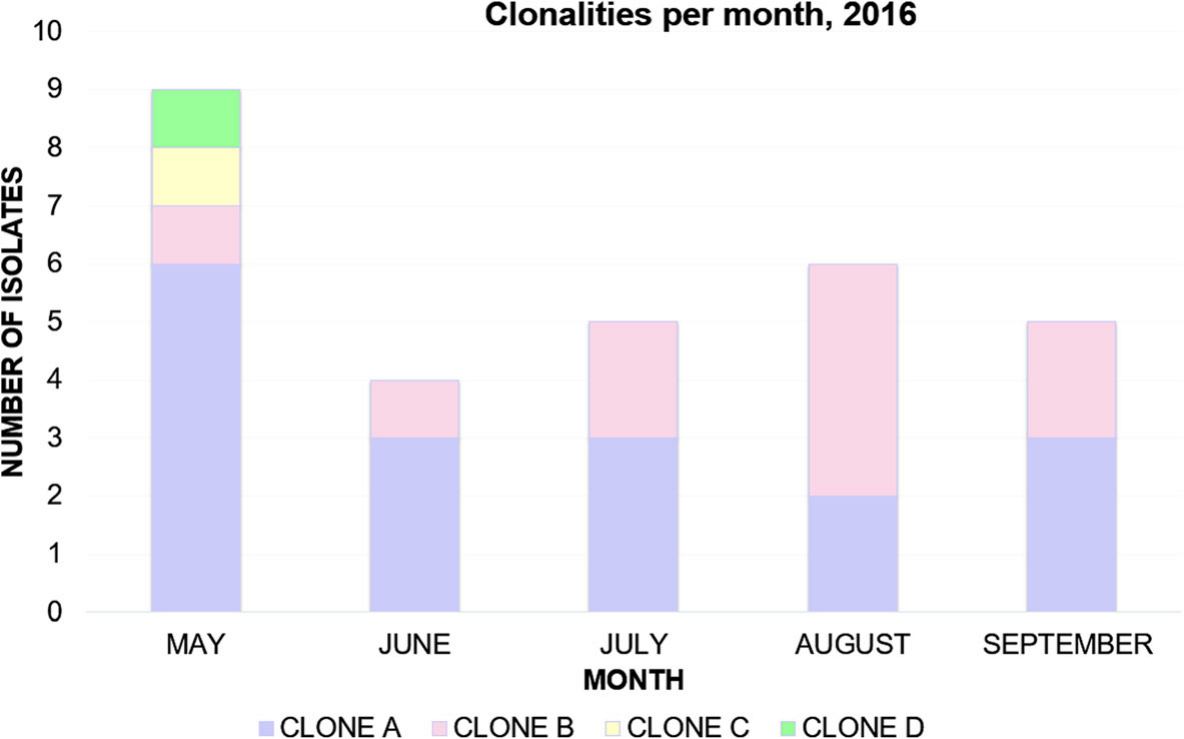
Clonal profiles identified throughout May to September 2016. Clonal profile “A” occurred most frequently in clinical isolates during May to September. Profile “B” was the most identified in August; however, profile “A” once again became recognised in September as the most documented profile in VR*Efm* isolates. Number of isolates collected per month according to their clonal profile are shown

Molecular typing was based on VNTR-1, VNTR-7, VNTR-8, VNTR-9 and VNTR-10 grouping and was performed for the 33 recovered VR*Efm* isolates (including environmental samples). Examples of the different VNTRs are shown in Additional file 2: Figure S2. Four clonal profiles (A, B, C and D) were identified from the isolates. The most identified profile for all clinical strains (29 isolates) was “A”, in 17 of the strains. The second was profile “B” in 10 of the strains, and “C” and “D” profiles were identified in one strain, each. Outbreak isolates were classified as 9 (69.2%) belonging to clonal profile “A”, 2 (15.4%) to profile “B”, whilst clonalities “C” and “D” were each recognised in just one isolate (7.7%). Profile “C” was also recovered from an environmental surface (bed-rail). Profile “D” was only identified in one external source isolate (having the lowest vancomycin-resistance level and being susceptible to teicoplanin and ampicillin). Only profiles “A” and “B” were identified in the post-outbreak isolates (16 strains), 8 for “A” (50%) and 8 for “B” (50%). No environmental study was implemented during this period. (Table 2a and b). Figure 3 shows the number of isolates displaying each clonal profile, distributed per month from May to September 2016.

### Transmission analysis

A TPS algorithm (previously described for *Pseudomonas aeruginosa*) [27] was adapted to assess transmission routes (direct transmission or cross-transmission) in the 16 outbreak patients only; data collected from these 16 patients related to the VR*Efm* outbreak was analysed and epidemiological criteria (possible and probable) were applied to all of them; however, genetic relatedness (predicted transmission) was evaluated in 13 out of the 16 samples as no isolates were recovered from the first 3 outbreak patients.

**Fig. 4:**
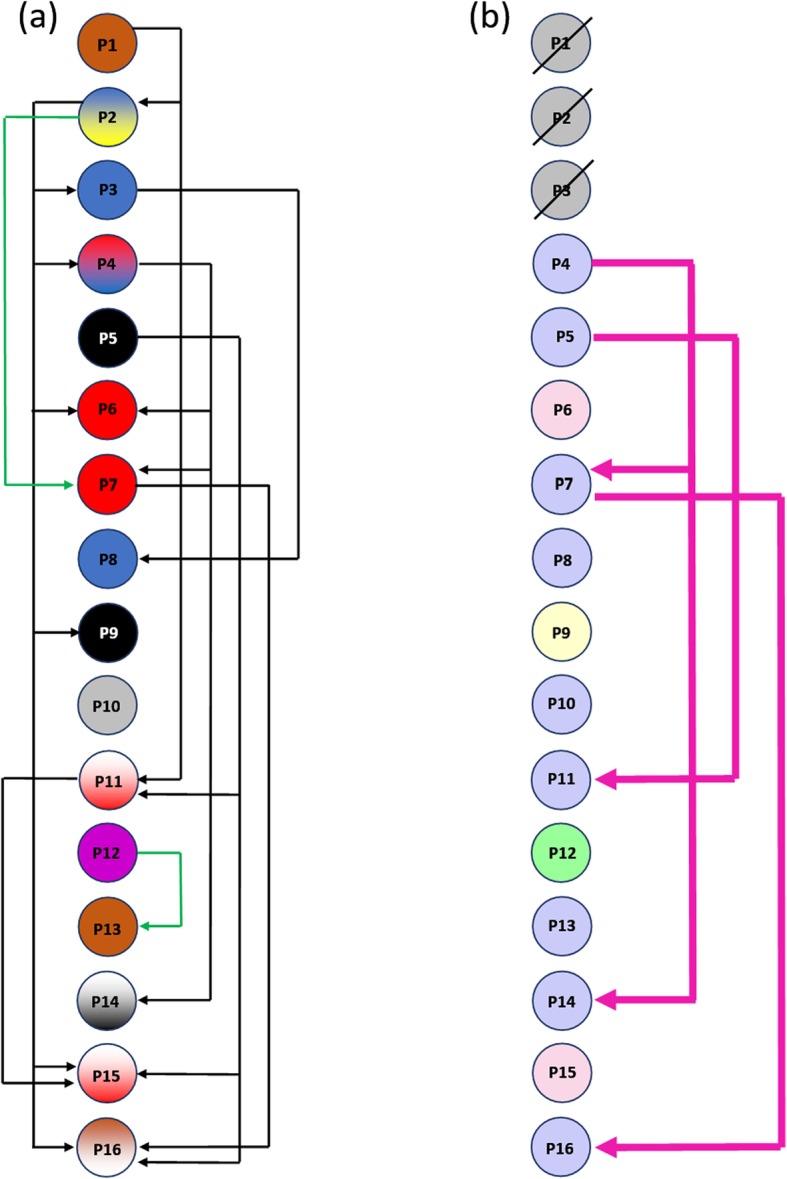
Estimated transmission routes for the 16 outbreak patients using the modified time-place-sequence (TPS) algorithm. Arrow colours reflect the estimated transmission probability between two patients. Criteria 1–4 are described briefly as: criterion 1, same ward, same time; criterion 2, same room consecutively within 14 days; criterion 3, same room, same time; and criterion 4, close genetic relatedness. Each patient is outlined as a circle. Figure 4(a) shows the probability of transmission based on the three epidemiological criteria alone: criterion 1 (black arrows), criterion 3 (green arrow), no patients fulfilled criterion 2. The colour of the circle indicates the patient’s location at the date of first VR*Efm* isolation. White stands for ward 2 (ICU), yellow stands for ward 3 (Intermediate Care Unit), blue stands for general ward 4, red stands for general ward 5, black stands for general ward 6, green stands for general ward 7, grey stands for general ward 8, and purple stands for the Emergency Room (ER). In the case of 4 patients (P2, P4, P11, P16), their location at the date of first VR*Efm* isolation varied within the same day. Similarly, for patients P14 and P15, they had not completed 24 h of stay in a ward when the fist isolation was detected; all of them are thus represented with 2 colours. Figure 4(b) shows the probability of transmission based on the three epidemiological criteria in combination with the genetic relatedness criterion (pink arrows). The colour of the circle indicates an isolate’s clonal profile. Purple is clonal profile “A”, pink profile “B”, yellow “C” and Green “D”

TPS algorithm analysis was used to track transmission routes in the outbreak patients. The epidemiological module was run for all 16 patients whilst epidemiological + genetic relatedness modules were run for 13 of them. P1 was the first patient identified during the outbreak (index case), but P2 was the estimated case who infected the greatest number of patients according to patient flow tracking. Using the adapted algorithm was aimed at investigating whether genetic relatedness based on VNTRs grouping (clonalities) could improve transmission route tracing compared to just conventional epidemiological surveillance, typically involving time (epidemic curve), space (geographic distribution) and person (patient characteristics) [29]. Figure 4a shows possible (criterion 1) and probable (criteria 2 and 3) transmission, and Fig. 4b shows predicted transmission (criterion 4 + 1 or 2 or 3). Four transmissions which had been classified as possible based on epidemiological criteria alone could be predicted with high probability by also applying genetic criterion 4. Looking at these four cases, it is worth noting that patients stayed on the same ward at the same time, but not in the same room (P4 likely transmitted his strain to P7 and P14; P5 likely transmitted his strain to P11; and P7 likely transmitted his strain to P16). Estimated transmission via healthcare personnel was thus likely (Fig. 4b). Patient 9 became infected during her hospital stay, having a different clonal profile, and was not noticeably exposed to previously infected patients (Fig. 4b).

Considering the high mobility between wards and floors that the patients had, it was difficult to trace the transmission between them. Some patients changed rooms four or more times during the outbreak, so representing an epidemiological curve including rooms was difficult, but it was constructed by wards (Additional file 3: Figure S3). Nevertheless, if a detailed map of where each patient was at each time is required, a spreadsheet depicting this can be found as Additional file 4: Table S1.

## Discussion

This study’s outcomes meant that our goal of describing VR*Efm* nosocomial spread in a large hospital setting was achieved. The Colombian National Institute of Health uses repetitive element sequence-based PCR (rep-PCR) to assess infectious disease outbreaks and determine strain clonality; however, it has been reported that this is not the most accurate method for such determination [33, 34, 40, 41].

Particularly interesting findings concerned phenotype/genotype correlations, such as teicoplanin- and ampicillin-susceptible phenotype expression with *vanA* gene identification in one isolate, and a lack of *vanA* gene detection in three high-level vancomycin-resistant isolates. Some of these molecular and microbiological discrepancies have been determined as being due to a lack of *van* gene identification and two *van* genes being identified in the same isolate [42–45]. Chronological clonal profile follow-up (Fig. 3) suggested a transitional spread from profile “A” to profile “B”, returning to a higher prevalence of “A” at the end of the study. The persistence of clonal profile “A” being identified over the 5-month period indicated the risk of a second latent *Enterococcus faecium* outbreak. This risk was successfully dealt with since strategies aimed at preventing healthcare-associated infections and the early identification of an increased number of isolates were maintained. Prospective investigation of these isolates confirmed the multisource VR*Efm* outbreak and the insidious pattern of clonal profile “A” which was also observed in two out of the three environmental samples collected (Table 2b).

Most patients were not directly exposed to each other but had been hospitalised in wards on the same floor. As clonal profiles “C” and “D” did not spread, this also supports the idea that suitable hospital wardroom cleaning was insisted on, thereby ensuring that these strains did not keep on spreading; an additional explanation for this spreading behaviour between strains, could be the presence of different clonal population types, as most hospital-derived isolates worldwide are linked to a polyclonal subpopulation (particularly MLST sequence type 17 (ST17), ST18, ST78 and ST192, which were previously designated clonal complex CC17 [18, 31, 46, 47]. The described transmission route (criterion 1) suggested cross-contamination between patients via healthcare workers, possibly due to a lack of adherence to epidemiological sanitary measures, such as hand washing/hand hygiene and the appropriate use of medical devices.

This study has shown how our approach to the VR*Efm* outbreak in the Méderi hospital, involving an integrated molecular and epidemiological model, enabled accurately evaluating the effectiveness of the epidemiological measures and identifying the main cause related to the spread of VR*Efm*. The main limitation of this study lies in the lack of systematic rectal screening for VRE, as unknown carriers are a hidden reservoir for the transmission and dissemination of VRE and colonisation is much more frequent than infection.

## Conclusions

The benefits of real-time combined clinical and molecular epidemiological models, as previously described in different infectious disease outbreak investigations [27, 48–52], contribute towards introducing/enforcing more efficient outbreak control strategies. Preventing such kind of hospital threats could depend on reinforcing one of the most ancient and cost-effective epidemiological interventions: hand-washing (as in our case).

## Additional files

Additional file 1: **Figure S1.** VR*Efm* outbreak response and incident management. The Figure shows the departments involved and interventions made during the outbreak (TIF 132 kb)

Additional file 2: **Figure S2.** Molecular typing of VR*Efm* isolates. PCR amplification of the most frequently found clonal profile “A” in VR*Efm* isolates. Lanes: M, DNA size marker; 1, VNTR-1; 2, VNTR-7; 3, VNTR-8; 4, VNTR-9; 5, VNTR-10. Samples were processed as previously described by Top et al. [34], with some minor modifications. (TIF 5110 kb)

Additional file 3: **Figure S3.** Epidemiological curve depicting VR*Efm* acquisition according to patient location by wards. (TIF 257 kb)

Additional file 4: **Table S1.** Inpatient tracking within de hospital. X-axis corresponds to the days of month, starting from April 1st to June 30th. Y-axis corresponds to wards or floors and bed number. Only beds occupied by patients from the outbreak were included for each ward. Patients are represented by numbers from 1 to 16. Background colours represents clonal profiles, as described in Table 2 and Fig. 4. For the first three patients, the clonal profile was not determined, so they are shown in grey colour. Lighter shade of colours corresponds to the period in which the isolate had not yet been identified. Black colour indicates the sampling date from which the first VR*Efm* was confirmed. Darker shade of colours indicates the period after VR*Efm* confirmation. Red colour points out dates in which patients shared a room. (XLSX 29 kb)

## Abbreviations

AMR: Antimicrobial resistance
CCU: Coronary care unit
CLSI: Clinical Laboratory Standards Institute
*Efm*: *Enterococcus faecium*
HAIs: Healthcare-associated infections
ICU: adult intensive care unit
IMCU: adult intermediate care unit
MICs: Minimum inhibitory concentrations
MLVA: Multilocus variable number tandem-repeat analysis
NICU: Neonatal intensive care unit
rep-PCR: repetitive element sequence-based PCR
SHEA: Society for Healthcare Epidemiology of America
TPS: Time-place-sequence
VNTR: Variable-number tandem-repeat
VRE: Vancomycin-resistant *Enterococci*
VR*Efm*: Vancomycin-resistant *E. faecium*
