# Supplementary figures and images for: An epidemiological and molecular study regarding the spread of vancomycin-resistant Enterococcus faecium in a teaching hospital in Bogotá, Colombia 2016
Source: BMC Infect Dis. 2019 Mar 15;19:258. doi: 10.1186/s12879-019-3877-7 (PMC6419805; doi:10.1186/s12879-019-3877-7)

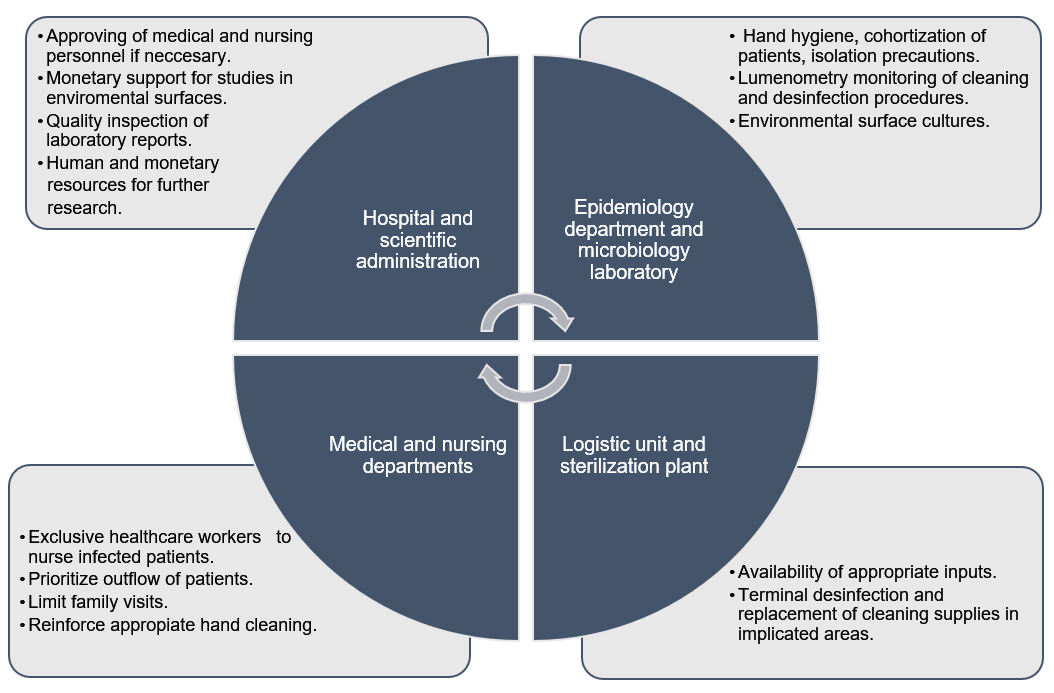

Supplement: Supplementary file 1 — Figure S1. VREfm outbreak response and incident management. The Figure shows the departments involved and interventions made during the outbreak (TIF 132 kb) [file 12879_2019_3877_MOESM1_ESM.tif]

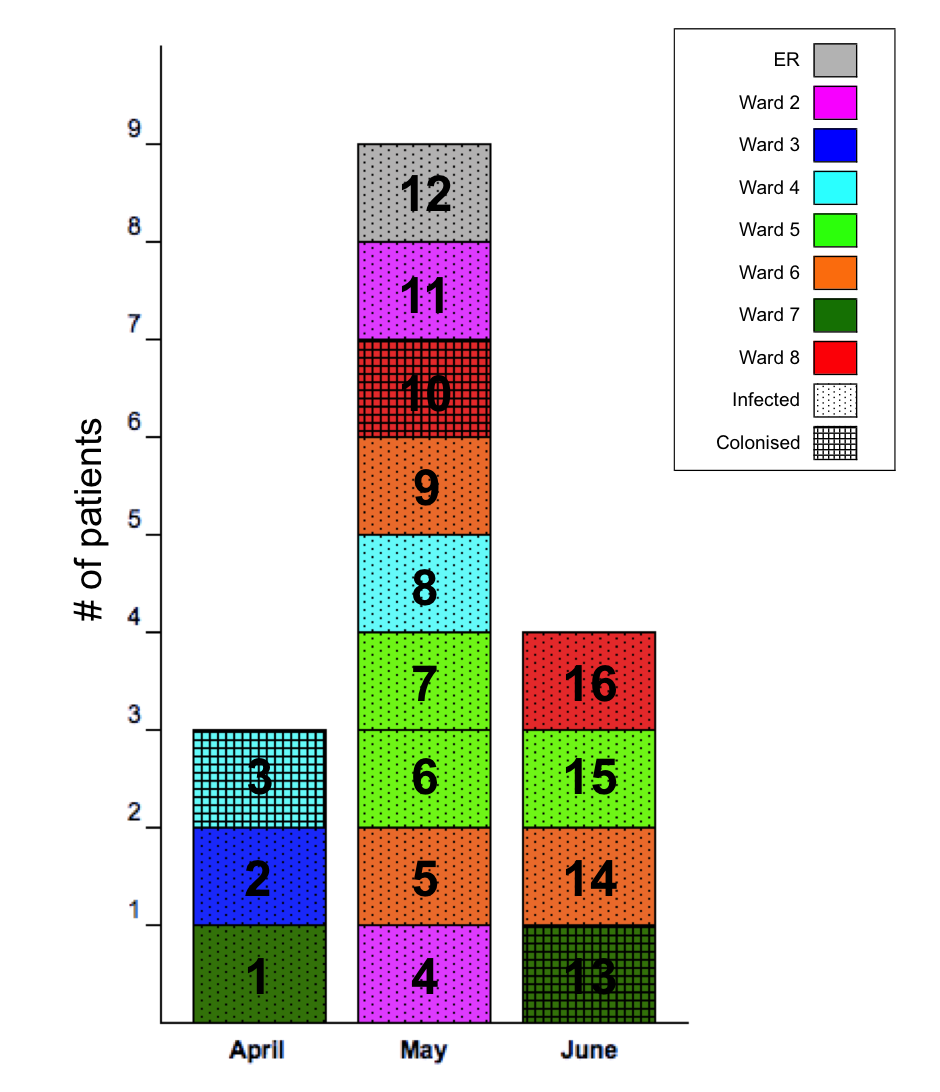

Supplement: Supplementary file 3 — Figure S3. Epidemiological curve depicting VREfm acquisition according to patient location by wards. (TIF 257 kb) [file 12879_2019_3877_MOESM3_ESM.tif]
